# The Dual Role of miR-186 in Cancers: Oncomir Battling With Tumor Suppressor miRNA

**DOI:** 10.3389/fonc.2020.00233

**Published:** 2020-03-05

**Authors:** Ying Xiang, Qing Tian, Li Guan, Shuai-shuai Niu

**Affiliations:** ^1^Laboratory of Oncology, Center for Molecular Medicine, School of Basic Medicine, Health Science Center, Yangtze University, Hubei, China; ^2^Department of Cell Biology and Genetics, School of Basic Medicine, Health Science Center, Yangtze University, Hubei, China; ^3^The First School of Clinical Medicine, Health Science Center, Yangtze University, Hubei, China

**Keywords:** miRNA, cancer, oncomir, tumor suppressor miRNA, dual role

## Abstract

MicroRNAs (miRNAs) are a class of small non-coding RNAs which regulate gene expression at post-transcriptional level. Alterations of miR-186 expression were demonstrated in numerous cancers, shown to play a vital role in oncogenesis, invasion, metastasis, apoptosis, and drug resistance. MiR-186 was documented as a tumor suppressor miRNA in the majority of studies, while conflicting reports verified miR-186 as an oncomir. The contradictory role in cancers may impede the application of miR-186, as well as other dual-functional miRNAs, as a diagnostic and therapeutic target. This review emphasizes the alterations and functions of miR-186 in cancers and discusses the mechanisms behind the contradictory findings. Among these, target abundance and dose-dependent effects of miR-186 are highlighted. The paper aims to review the challenges involved in developing diagnostic and therapeutic strategies for cancer treatment based on dual-functional miRNAs.

## Introduction

MicroRNAs (miRNAs), about 22 nucleotides in length, are non-coding RNAs which regulate gene expression at a post-transcriptional level, mediating they target mRNAs translation inhibition or degradation via binding to the 3′-untranslated regions (UTR) (1). MiRNAs are essential in a number of pathways related to physiological and pathological processes, including cell cycle, proliferation, migration, and apoptosis. Recently, dysregulation of miRNAs in cancers has become a focus of research (2).

MiR-186 (also known as miR-186-5p) was first identified from the human Saos-2 cell line in 2003 (3). The MiR-186 gene is located on chromosome 1, within intron 8 of the ZRANB2 (zinc finger RANBP2-type containing 2) gene. (4). ZRANB2, a widely expressed spiceosomal protein found in a variety of tissues, is important for alternative splicing of transcripts. Antoniou et al. found that miR-186 displayed a similar expression profile to *ZRANB2* during differentiation of muscle cells. That is to say, miR-186 was transcribed with its host gene *ZRANB2* synchronously (5). The level of miR-186 in cancer cells may be dysregulated at a transcriptional level by the DNA methylation stage of the host gene promoter or the transcription factors (which bind with the promoter or other *cis*-elements). More commonly, it may be regulated by LncRNAs post-transcriptionally. It was reported that LncRNA PVT1 served as a sponge containing a complementary nucleotide sequence to miR-186, downregulating miR-186 expression in gastric cancer cells (6), liver cancer cells (7), and glioma vascular endothelial cells (8), so that it attenuated the function of miR-186.

**Figure 1:**
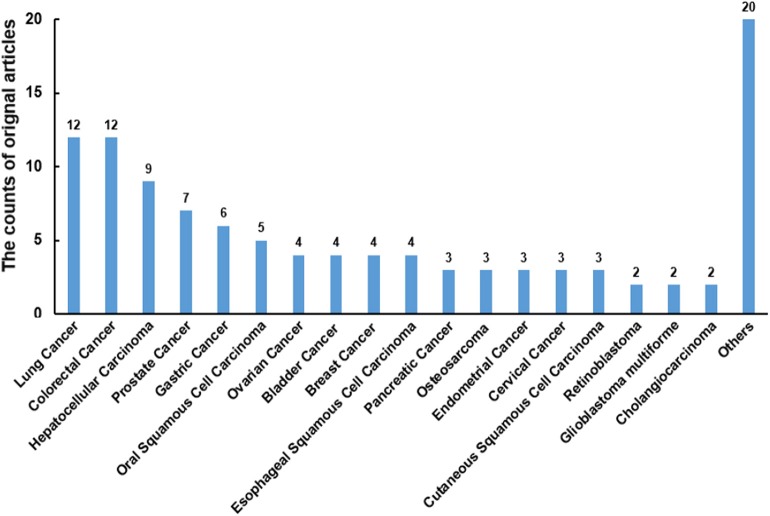
The counts of original articles about miR-186 in various malignancies from 2008 to 2019 year in PubMed.

There were 108 original articles about “miR-186” in the field of cancer research from 2008-2019 year in PubMed, and a large proportion of these publications emerged during 2016-2019(85/108). Alterations of miR-186 expression were demonstrated in numerous cancers. The function of miR-186 was most investigated in lung cancer, colorectal cancer, hepatocellular carcinoma, prostate cancer, and gastric cancer (Figure 1). The majority of targets were revealed in lung cancer, hepatocellular carcinoma, and prostate cancer (Table 1), indicating that miR-186 may be applied as a potential therapeutic target in these cancers. In patients with non-small cell lung cancer (NSCLC) (14) and breast cancer (39), downregulation of miR-186 in tissues predicted a poor survival rate, suggesting miR-186 may act as a diagnostic and prognostic marker. MiR-186 was documented as a tumor suppressor miRNA in the majority of studies, while some reports verified miR-186 as an oncomir. The contradictory role in cancers may impede the application of miR-186 as a diagnostic and therapeutic target, and it is of great importance to explore the possible mechanisms behind the contradictory findings.

**Table 1:** Alterations of miR-186 and verified targets in cancers.

**Types of cancer/cells**	**Alteration of miR-186**	**Function of miR-186**	**Validated targets**	**References**
Endometrial cancer	Upregulated	Oncomir	P2RX7, FOXO1	(9, 10)
Cutaneous squamous cell carcinoma	Upregulated	Oncomir	APAF1, RETREG1	(11, 12)
Cervical cancer	Upregulated	Oncomir	P2RX7	(9)
Non-small cell lung carcinoma	Upregulated	Oncomir	PTEN	(13)
Non-small cell lung carcinoma	Downregulated	Tumor suppressor miRNA	CCND1, CDK2, CDK6, SIRT6, ROCK1, MAP3K2, YY1, Cdc42, CDK1	(14–19) (20)
Prostate cancer	Upregulated	Oncomir	AKAP12	(21)
Prostate cancer	Downregulated	Tumor suppressor miRNA	Twist 1, GOLPH3, YY1,CDK6	(22–24)
Bladder cancer	Upregulated	Oncomir	RETREG1	(25)
Bladder cancer	Downregulated	Tumor suppressor miRNA	NSBP1, VEGF-C	(26, 27)
Colorectal cancer	Upregulated	Oncomir	FAM134B	(28)
Colorectal cancer	Downregulated	Tumor suppressor miRNA	ZEB1	(29)
Pancreatic ductal adenocarcinoma	Upregulated	Oncomir	NR5A2	(30)
Pancreatic cancer	Downregulated	Tumor suppressor miRNA	YAP1	(31)
Hepatocellular Carcinoma	Downregulated	Tumor suppressor miRNA	YAP1, HMGA2, MAP4K3, ROCK1, MCRS1	(7, 32, 33) (34–36)
Gastric cancer	Downregulated	Tumor suppressor miRNA	Twist1, NEK2	(37, 38)
Breast cancer	Downregulated	Tumor suppressor miRNA	Twist1	(39)
Cholangiocarcinoma	Downregulated	Tumor suppressor miRNA	Twist1	(40)
Ovarian cancer	Downregulated	tumor suppressor miRNA	PIK3R3	(41)
Glioma vascular endothelial cells	Downregulated	Tumor suppressor miRNA	Atg7, Beclin1	(8)
Glioma stem cells	Downregulated	Tumor suppressor miRNA	XIAP, PAK7	(42)
Glioblastoma multiforme	Downregulated	Tumor suppressor miRNA	FGF2, RelA	(43)
Pituitary tumors	Downregulated	Tumor suppressor miRNA	SKP2	(44)
Esophageal squamous cell carcinoma	Downregulated	Tumor suppressor miRNA	SKP2	(45)
Oral squamous cell carcinoma	Downregulated	Tumor suppressor miRNA	SHP2	(46)
Follicular thyroid carcinoma	Downregulated	Tumor suppressor miRNA	ST6GAL2	(47)
Multiple myeloma	Downregulated	Tumor suppressor miRNA	Jagged1	(48)
CML	Downregulated	Tumor suppressor miRNA	DDX43	(49)
Retinoblastoma	Downregulated	Tumor suppressor miRNA	DIXDC1, ATAD2	(50) (51)
Osteosarcoma	Downregulated	Tumor suppressor miRNA	TBL1XR1, FOXK1	(52) (53)
Renal Cell Carcinoma	Downregulated	Tumor suppressor miRNA	SENP1	(54)
Cisplatin-resistant glioblastoma cells	Downregulated	Reverse resistance	YY1	(55)
Cisplatin-resistant ovarian cancer/ cells	Downregulated	Reverse resistance	Twist1, ABCB1	(56, 57)
Taxol-resistant ovarian cancer cells	Downregulated	Reverse resistance	ABCB1	(57)
Paclitaxel-resistant non-small cell lung cancer	Downregulated	Reverse resistance	MAPT	(58)
MTX-resistant colorectal cancer	Downregulated	Reverse resistance	CPEB2	(59)

## Altered Expressions of miR-186 in Different Cancers

Alterations of miR-186 expression were demonstrated in numerous cancer tissues or cell lines, which played a vital role in oncogenesis, invasion, metastasis, apoptosis, and drug resistance. In endometrial cancer tissues and squamous cell carcinoma tissues, miR-186 was significantly upregulated in comparison with relative non-cancerous tissues. MiR-186 was verified to play a role as an oncomir which enhanced proliferation and migration and repressed apoptosis. However, the majority of studies showed that miR-186 was decreased in solid cancers, including gastric cancer, oral squamous cell carcinoma, hepatocellular carcinoma, breast cancer, glioblastoma multiforme, esophageal squamous cell carcinoma, pituitary tumors, follicular thyroid carcinoma, retinoblastoma, osteosarcoma, cholangiocarcinoma and blood malignancy multiple myeloma, and CML, and that miR-186 served as a tumor suppressor miRNA. Notably, there were conflicting reports of miR-186 alterations in NSCLC, bladder cancer, prostate cancer, colorectal cancer, and pancreatic cancer (Table 1).

NSCLC, which accounts for 70–80% of lung cancer, is one of the most common malignancies globally. Feng et al. confirmed miR-186 significantly upregulated in lung adenocarcinoma samples and cells, in comparison with the adjacent non-cancerous samples and normal lung epithelial cells BEAS-IB (13). However, several groups reported that the levels of miR-186 were markedly decreased in NSCLC samples and cells (14–20). Prostate cancer is the most common non-skin cancer in males, with there being ~1.6 million cases worldwide annually (60). One report showed the levels of miR-186 were increased in metastatic prostate cancer cells compared to normal prostate epithelial cells RWPE1 (21), while two groups demonstrated that miR-186 was downregulated in prostate cancer tissues and cells (22, 23). Bladder cancer is the seventh most common cancer in females and the fourth most common in males worldwide (61). Yang and his colleagues concluded that the upregulation of miR-186 in bladder cancer tissue samples in comparison with corresponding para-cancerous samples after a TCGA data analysis, further verified upregulation of miR-186 in bladder cancer cells in comparison with normal human uroepithelial cells (25). However, two groups reported downregulation of miR-186 in bladder cancer tissues and cells (26, 27). Colorectal cancer is the third most common cancer in the world (62). Islam et al. revealed miR-186 was significantly upregulated in colorectal cancer tissues and cells, in comparison with the corresponding normal tissues and normal colonic epithelial cells (28), while Li et al. reported a significant downregulation of miR-186 in colorectal cancer tissues and cells (29). Pancreatic ductal adenocarcinoma (PDAC) is one of the deadliest malignancies (63). Zhang et al. found upregulation of miR-186 in PDAC tissues and cells related to the adjacent normal pancreatic samples and human pancreatic ductal epithelium cells (30), whereas Niu et al. found miR-186 was significantly downregulated in pancreatic cancer tissues (31).

## The Dual Role of miR-186 in Cancers

The consequences of altered expressions of miR-186 were related to enhanced or repressed cell proliferation, invasion, metastasis, and apoptosis, as well as drug resistance in cancers. MiR-186 may serve as an oncomir or a tumor suppressor miRNA. Researchers validated 48 targets of miR-186 in 25 types of cancer tissues or cancer cells (Table 1). The expression profiles of the majority of targets were verified to be negatively correlated with miR-186, such as Twist1, RETREG1, ROCK1, GOLPH3, NSBP1, APAF1, SHP2, and so on.

### miR-186 as an Oncomir

**Figure 2:**
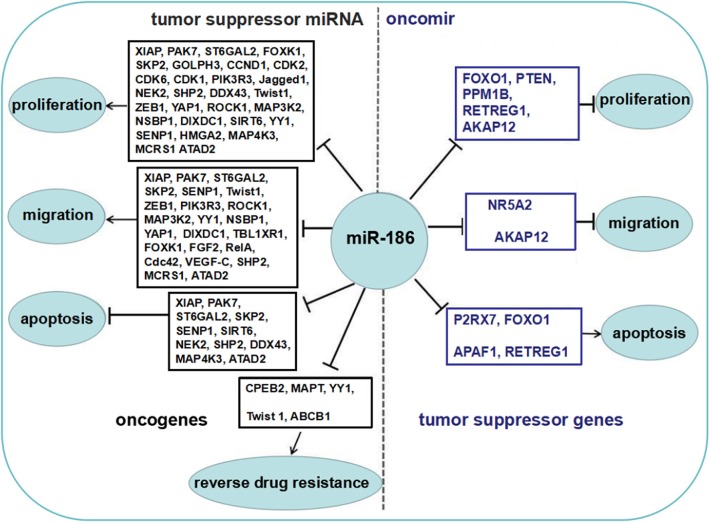
The role of miR-186 as an oncomir or a tumor suppressor miRNA. Blue boxes display tumor suppressor genes among the targets of miR-186, black boxes display oncogenes among the targets of miR-186. MiR-186 is involved in regulation of cell proliferation and growth, migration and invasion, apoptosis, and resistance to chemotherapeutic drugs by suppressing multiple targets.

Some reports showed that miR-186 was upregulated in several cancers, and served as an oncomir, which both promoted cell proliferation and migration, and inhibited apoptosis by repressing several targets (Figure 2).

In endometrial cancer tissues and squamous cell carcinoma tissues, miR-186 served as an oncomir by suppressing targets P2RX7, FOXO1, APAF1, and RETREG1. The P2X7 receptor (P2RX7) was a coordination channel binding on the membrane, activation of which induced stomata formation on the cell membrane and mediated the apoptosis of epithelial cells. Zhou et al. reported miR-186 at a higher level in endometrial cancer tissues induced the degradation of P2RX7 (9). Myatt et al. reported that miR-186 repressed FOXO1 expression by targeting the 3′-UTR of FOXO1, thus promoting proliferation and viability of endometrial cancer cells. FOXO1, as a tumor suppressor and a transcription factor of the FOXO family, activated the transcription of FOXO downstream targets, some of which displayed vital roles in cell cycle and apoptosis (10). APAF1, a critical component of the apoptosome to promote endogenous apoptotic process, was verified to be a functional target of miR-186 in tissues and cells of squamous cell carcinoma (cSCC). Enforced overexpression of miR-186 in cSCC cells significantly increased cell growth, invasion, and migration, and apoptosis was significantly increased when miR-186 expression was inhibited (11). RETREG1, also known as FAM134B, was another target of miR-186 in increasing proliferation and inhibiting apoptosis in cSCC. RETREG1 contributed to the normal functioning of endoplasmic reticulum (ER). Inhibition of RETREG1 led to the errors in folding of proteins in ER, resulting in impaired protein balance and disease (12).

In the five types of controversial cancers (NSCLC, bladder cancer, prostate cancer, colorectal cancer, pancreatic cancer), several reports showed miR-186 served as an oncomir by suppressing targets PTEN, PPM1B, RETREG1, AKAP12, or NR5A2. Feng et al. confirmed miR-186 significantly enhanced cell proliferation and migration of lung adenocarcinoma by targeting PTEN (phosphatase and tensin homolog) directly (13). PTEN, serving as a tumor suppressor, negatively regulated the PI3K signaling pathway which contributed to a variety of cellular processes, such as cell differentiation, proliferation, survival, motility, and invasion (64). Yang and his colleagues verified that upregulation of miR-186 significantly enhanced the growth rates of bladder cancer cells by targeting PPM1B, which consequently promoted expression of p21Cip1 and p27Kip, while decreasing cyclin D1 expression level, which facilitated G1-S phase transition (25). Islam et al. revealed that miR-186 increased proliferation and migration of colorectal cancer cells by targeting RETREG1 (28), which regulated proteostasis by the turnover of endoplasmic reticulum. AKAP12, a scaffold protein, regulated cytoskeletal remodeling and mitogenic signals by combining multiple signal molecules. AKAP12 was a verified target of miR-186 (65). Jones et al. found downregulation of endogenous miR-186 with an inhibitor upregulated the expression of AKAP12 in prostate cancer cells, thus repressing anchorage-independent growth as well as invasion of prostate cancer cells (21). Zhang et al. found overexpression of miR-186 enhanced proliferation and migration of pancreatic ductal adenocarcinoma (PDAC) cells via targeting NR5A2 (30). NR5A2 (also called LRH-1), a transcription factor abundant in the cytoplasm and nucleus, interacted with both Hedgehog and Wnt/β-catenin. When NR5A2 interacted with β-catenin, it affected the expression of cell cycle genes such as CCND1 and CCNE1 genes, as well as MYC genes (66).

### miR-186 as a Tumor Suppressor miRNA

The majority of reports showed that miR-186 was downregulated in a variety of cancers and served as a tumor suppressor miRNA which repressed proliferation and migration, and facilitated apoptosis by targeting multiple targets (Figure 2).

Epithelial-mesenchymal transition (EMT) describes the change from epithelial cells (immotile) to mesenchymal cells (motile), with gene expression and phenotypic alterations facilitating tumor metastases. EMT is vital for tumor progression, as well as chemotherapeutic resistance (67). Twist 1 (the Twist family bHLH transcription factor 1), a well-known transcription factor in regulation of EMT (68), was shown to be a target of miR-186 in several types of cancers such as prostate cancer (24), gastric cancer (37), breast cancer (39), and cholangiocarcinoma (40). Overexpression of miR-186 in these cancer cells inhibited proliferation, EMT, and migration via suppressing Twist 1. YAP1 was one important effector of the Hippo pathway and had crosstalk with other pathways, regulating the expression of genes which were involved in proliferation and EMT (69). By targeting YAP1, miR-186 inhibited proliferation, migration, and invasion of pancreatic cancer (31) and hepatocellular carcinoma cells (32). Moreover, miR-186 repressed invasion and migration by directly targeting TBL1XR1 and FOXK1 in osteosarcoma cells (52, 53), or targeting FGF2 and RelA in glioblastoma multiforme cells (43). MiR-186 inhibited proliferation of human pituitary tumor cells through targeting SKP2, thus upregulating p27^Kip1^ expression, a well-known negative regulator of G1 cell cycle progression (44). Jagged1 (JAG1), as a member of oncogenes, activated the Notch signal pathway related to tumorigenesis for some types of cancers (70). MiR-186 repressed cell proliferation by targeting JAG1 in multiple myelomas (48), or targeting HMGA2 in hepatocellular carcinoma cells (33). Additional targets including MCRS1 (which promoted the nuclear β-catenin accumulation and activated Wnt/β-catenin signaling) in hepatocellular carcinoma cells (36), PIK3R3 (a regulatory subunit of PI3K) in epithelial ovarian cancer cells (41), DIXDC1 in retinoblastoma cells (50), and SENP1 in renal cell carcinoma cells (54), were involved in the inhibitory effects of miR-186 on cell proliferation and invasion.

XIAP (X-linked inhibitor of apoptosis, a class of anti-apoptotic proteins), and PAK7 (also known as PAK5, an evolutionarily conserved serine/threonine protein kinase), were highly expressed in glioma cells. MiR-186 inhibited the proliferation, migration, and invasion of glioma stem cells and promoted apoptosis via targeting XIAP and PAK7, thus regulating the expression levels of downstream target proteins such as caspase 3, BAD, cyclin D1, and MARK2 (42). Moreover, miR-186 suppressed cell proliferation and induced apoptosis by targeting either: MAP4K3 in hepatocellular carcinoma (34), NEK2 in gastric cancer cells (38), SKP2 in esophageal squamous cell carcinoma cells (45), SHP2 in oral squamous cell carcinoma cells (46), or targeting DDX43 (also known as HAGE, a cancer/testis antigen) in CML cells (49). In addition, miR-186 suppressed the proliferation, migration, invasiveness, and angiogenesis by targeting ST6GAL2 in follicular thyroid carcinoma cells (47), or targeting ATAD2 in retinoblastoma cells (51), and inhibited autophagy by targeting the autophagy-related proteins Atg7 and Beclin1 in glioma microvascular endothelial cells (8).

In the five types of controversial cancers, there were many reports that miR-186 served as a tumor suppressor miRNA by target multiple oncogenes, which was contradictory to the previous statements. In NSCLS, miR-186 inhibited cell proliferation by targeting CCND1 (coding cyclins D1), CDK2, CDK6 (14), CDK1 (20), SIRT6 (15), and ROCK1 (16), inhibited migration and invasion via targeting ROCK1 (16), MAP3K2 (17), YY1 (18), and Cdc42 (19), and induced cell apoptosis via targeting SIRT6 (15). The cyclin-CDK complexes promotes cellular proteins phosphorylation, then drive the progress of cell cycle, which is essential in tumorigenesis (71). ROCK1 (Rho-associated protein kinase 1), could promote actin cytoskeleton reorganization during cell motion and invasion (72). MAP3K2 (Mitogen-activated protein kinase kinase kinase 2), could enhance the MAPK signal pathway by activation of c-JNK and ERK5. YY1 (Yin Yang 1), a relatively conserved transcription factor, is essential in embryonic development, differentiation, proliferation, migration, and invasion of solid tumors. Cdc42, belonging to the Rho-GTPase family, is vital for the establishment and persistence of cell migration. In prostate cancer, miR-186 repressed cell proliferation via targeting YY1 and CDK6 (23) or GOLPH3 (22). Downregulation of GOLPH3 enhanced p21 expression and repressed the expression of Cyclin B1 and CDK1/2, thus blocking G1/S transition (73). MiR-186 repressed invasion and migration of prostate cancer cells by targeting Twist 1 (24). In bladder cancer, miR-186 markedly repressed cell proliferation and metastasis by targeting NSBP1 (also known as HMGN5) (26), which can bind to nucleosomes with its nucleosomal-binding domain to make chromatin unfold, regulating the expression of many genes (74). VEGF-C was also a target through which miR-186 repressed invasion and migration of bladder cancer cells (27). In colorectal cancer, Li et al. reported that miR-186 repressed cell proliferation, EMT, and migration by targeting ZEB1, a key member of the ZEB family, which were important in regulation of EMT in various cancer cells (29). In pancreatic cancer, Niu et al. verified that miR-186 inhibited cell proliferation, migration, and invasion by targeting YAP1 (31).

### miR-186 in Reversal of Drug Resistance

Chemotherapy is a fundamental treatment for cancer. However, drug resistance makes it not as efficient as expected. Cisplatin, taxol, and methotrexate (MTX) are common drugs used in chemotherapy. MiR-186 was downregulated in drug resistant cancer tissues and cells, including glioblastoma cells with cisplatin resistance, ovarian cancer tissues and cells with cisplatin resistance, ovarian cancer cells and NSCLC tissues with taxol resistance, and colorectal cancer tissues with MTX resistance. miR-186 was also observed to reverse resistance to cisplatin, taxol, and MTX (Table 1). MiR-186 increased cisplatin sensitivity by degrading YY1 in glioblastoma cells U87MG-CR (55). Overexpression of miR-186 in cisplatin-resistant cells of ovarian cancer induced the reversal of the EMT phenotype, cell cycle arrest, and cell apoptosis enhancement, so that the sensitivity to cisplatin was increased by miR-186. Twist 1 and ABCB1 were two functional targets of miR-186 in reversal of cisplatin resistance (56, 57). Otherwise, miR-186 was downregulated in ovarian cancer cells A2780/Taxol and in NSCLC tissues which were resistant to paclitaxel. Overexpression of miR-186 sensitized A2780/Taxol cells to taxol by targeting ABCB1 (57), as well as sensitized NSCLC cells A549 and H1299 to paclitaxel *in vitro* and in A549 xenografts by targeting MAPT (microtubule associated protein tau), which promoted microtubule assembly and leads to microtubule stabilization, by binding to the surfaces of microtubules outside and inside, interfering with the action of taxanes since they also bind to the inside surfaces (58). Moreover, miR-186 was decreased in MTX-resistant colorectal cancer. Overexpression of miR-186 promoted colorectal cancer cells more sensitive to MTX by targeting CPEB2 (cytoplasmic polyadenylation element binding protein 2) (59).

All in all, miR-186 may serve as an oncomir or a tumor suppressor miRNA in cancers. The dual role of miR-186 and biological functions of the verified targets are summarized in Figure 2.

## The Mechanisms Behind the Contradictory Findings

### The Contradictory Alterations of miR-186

In NSCLS, bladder cancer, prostate cancer, colorectal cancer, and pancreatic cancer, it was controversial that the levels of miR-186 were higher or lower than the match-adjacent tissues. Some proved miR-186 was at a higher level, while others reported miR-186 was at a lower level in the same cancer. We consider the following two aspects may contribute to the conflict.

### The Amount of the Samples and the Heterogeneity

**Figure 3:**
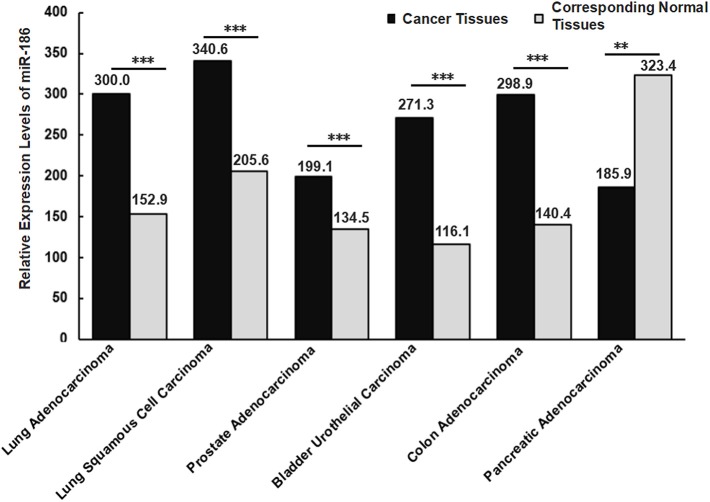
Relative expression levels of miR-186 in cancers (the data were obtained from starbase V2.0). Lung Adenocarcinoma (*N* = 512) vs. corresponding normal tissues (*N* = 20); Lung Squamous Cell Carcinoma (*N* = 512) vs. corresponding normal tissues (*N* = 20); Lung Squamous Cell Carcinoma (*N* = 475) vs. corresponding normal tissues (*N* = 38); Prostate Adenocarcinoma (*N* = 495) vs. corresponding normal tissues (*N* = 52); Bladder Urothelial Carcinoma (*N* = 408) vs. corresponding normal tissues (*N* = 19); Colon Adenocarcinoma (*N* = 450) vs. corresponding normal tissues (*N* = 8); Pancreatic Adenocarcinoma (*N* = 174) vs. corresponding normal tissues (*N* = 4). N indicates the number of tissue samples. ^***^P < 0.001, ^**^P < 0.01.

Islam et al. found that 70% (88/126) of colorectal cancer tissues exhibited high levels of miR-186, while 30% (38/126) exhibited low levels in patients with colorectal cancer. Nearly 69% (24/35) of lymphatic-infiltrated samples showed high levels of miR-186, while 31% (11/35) showed low levels of miR-186 (28). It revealed the alterations of miR-186 may be different due to the heterogeneity and the clinical stage of the patients even when they were affected by the same type of cancer. If we take a small number of samples, it could appear as though miR-186 is up-regulated in all cancer tissue samples in comparison with the matched-adjacent non-cancerous tissues, however the opposite results may be true when all of the samples are examined. That is, it is more reliable if more samples are analyzed. In order to increase the number of samples, we searched the expression levels of miR-186 in the five controversial cancers in the database (starbase V2.0), and we analyzed and summarized the data in a graphical form (Figure 3). It revealed the levels of miR-186 were increased in NSCLC (including lung adenocarcinoma and lung squamous cell carcinoma), prostate adenocarcinoma, bladder urothelial carcinoma, and colon adenocarcinoma tissues, and decreased in pancreatic adenocarcinoma tissues, in comparison with the corresponding normal tissues (Figure 3).

### Different Histological Subtypes

Different histological subtypes may contribute to contradictory alterations of miRNAs in a specific cancer. For example, Schmid et al. analyzed miR-34a expression in 133 tissue samples of epithelial ovarian cancer, and they found miR-34a was significantly downregulated in tissues of a histological subtype serous, endometrioid and mucinous ovarian cancer, while no significant alterations in tissues of a histological subtype clear cell ovarian cancer were found, in comparison with 8 healthy ovarian samples (75). This suggests that alterations of a certain miRNA varied with different histological subtypes even in a specific cancer. Similarly, diverse expression levels of miR-186 were observed in different histological subtypes of NSCLS, a higher level in lung squamous cell carcinoma compared with lung adenocarcinoma tissues (Figure 3). It is likely that the expression levels of miR-186 vary with histological subtypes in other cancers, such as bladder cancer, prostate cancer, colorectal cancer, and pancreatic cancer.

### The Contradictory Role of miR-186

In NSCLS, bladder cancer, prostate cancer, colorectal cancer, and pancreatic cancer, some studies proved that miR-186 served as an oncomir, while some reported that miR-186 served as a tumor suppressor in the same cancer type *in vitro* and *in vivo*. We consider the following three aspects may contribute to the contradictory role of miR-186.

### SNP Either in miRNA or in the 3′-UTR of Targets

MiRNAs exert their regulatory functions via binding to the 3′-UTR of target mRNAs. The binding affinity can be increased or decreased by single nucleotide substitutions either in DNA sequences for miRNAs or targets, which is called single-nucleotide polymorphisms (SNPs) (76). SNPs-rs66461782 in miR-186 was detected in patients with breast cancer (77). Not surprisingly, SNP may be detected in other types of cancer, so it may affect the function of miR-186 on its potential targets. The SNP related to target mRNAs was also one of concern to researchers. Rs1062577 was one of the miRNA-related ESR1 SNPs. The A allele, substitution for T at the site (rs1062577 A allele), attenuated the binding of miR-186 to ESR1 mRNA due to one hydrogen bond lost, then disturbed its negative regulatory effect of miR-186 on ESR1 transcripts (78). In a word, the function of miR-186 may be disturbed by SNP either in miR-186 or target mRNAs.

### The Targets Abundance and Network

Each miRNA regulates numerous target mRNAs by base pairing. MiR-186 can directly bind to target genes, affecting multiple pathways simultaneously. Moreover, these targets can also be regulated by other miRNAs besides miR-186. Although 48 targets of miR-186 have been verified to date, it is still difficult to reveal all the functional targets of miR-186 in cancers. Actually, the abundance of available targets may account for the varied outcomes of miRNAs. Aaron et al. found a significant correlation between the concentration of predicted targets of transfected miRNAs and the average expression of the target mRNAs. In the study, the predicted targets of miR-155, which were relatively few in number, were more downregulated than the targets of miR-128, which were more numerous (79). In addition, the abundance of a certain target may be involved in different outcomes of miRNAs. Myatt et al. verified FOXO1 was a direct target of 6 miRNAs (including miR-9, −27, −96, −153, −183, and −186) in endometrial cancer cells, and transfection of the anti-miRs effectively induced cell cycle arrest in Ishikawa cells (endometrial cancer cells with FOXO1 at low level), but no remarkable effects in HEC-1B cells (endometrial cancer cells with FOXO1 abundant) were found (10). Ding et al. verified that miR-204 inhibited the growth of prostatic adenocarcinoma cells, whereas it stimulated the growth of neuroendocrine-like prostate cancer cells via targeting XRN1, a dual regulator with variable abundance of proteins in these cells (80).

More importantly, if opposite functional molecules with different levels of abundance are the targets of a miRNA, then the ultimate effects are determined by the network of these targets. Niu et al. found miR-181a decreased the apoptosis of triple-negative breast cancer cells upon doxorubicin treatment through suppression of the pro-apoptotic protein BAX directly (81), while another report showed miR-181a enhanced adriamycin-induced apoptosis by targeting the anti-apoptotic protein Bcl-2 in low-invasive breast cancer cells (82). MiR-494 targeted both pro-apoptotic proteins (PTEN, ROCK1, and CaMKIIδ) and anti-apoptotic proteins (FGFR2 and LIF), and the ultimate consequence was cardioprotection (83). Obviously, many tumor suppressor genes and oncogenes were validated targets of miR-186 in cancers (Figure 2). We believe the abundance of these opposite functional targets may contribute to the contradictory findings of miR-186 in cancers.

### The Dose-Dependent Effects of miR-186

It was reported that the dose of miRNAs (miRNA abundance) affected their functions. Bu et al. found miR-34a distributed at high levels in differentiating progeny, whereas low levels of miR-34a demarcated self-renewing colon cancer stem cells. Moreover, both loss and gain of function of miR-34a altered the balance between self-renewal vs. differentiation (84). Yang et al. found that miR-181a significantly inhibited cell viability of breast cancer cells (MCF-7), dose-dependently. However, with a miR-181a dose higher than 50 nM, it promoted proliferation of several types of cancer cells rather than producing inhibitory effects (85). In addition, miRNA abundance affected the silencing of targets. Some target sites required higher miRNA concentration for silencing than others, since elevated miRNA levels could compensate for a lack of complementarity outside the seed (86). We suspect that different groups enhanced or inhibited miR-186 expression to varying degrees, leading to contradictory findings regarding the role of miR-186 in a certain cancer or cell lines.

**Figure 4:**
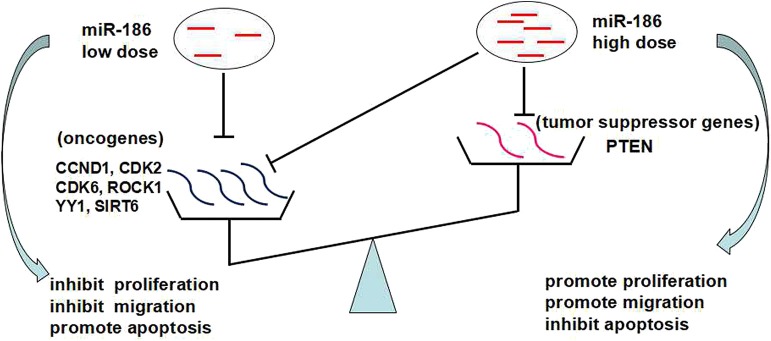
The dose-dependent effects of miRNAs and targets abundance may contribute to the contradictory role of miR-186.

Based on the above, we propose that the dose-dependent effects and targets abundance contribute to the contradictory role of miR-186: a low dose of miR-186 mainly targets oncogenes which is a more abundant target in cancers, playing a role as a tumor suppressor miRNA; a high dose (excessive) of miR-186 can also target tumor suppressor genes which is a less abundant target in cancer, playing a role as a tumor suppressor miRNA (Figure 4).

## Conclusion and Perspective

MiR-186 emerged as an essential miRNA involved in cell proliferation, apoptosis, migration, and invasion in various cancers. The majority of studies showed miR-186 serving as a tumor suppressor miRNA in variable cancers. Some studies verified that miR-186 played a role as an oncomir in endometrial cancer and cutaneous squamous cell carcinoma. However, there were conflicting reports in regards to NSCLC, bladder cancer, prostate cancer, colorectal cancer, and pancreatic cancer. It was controversial as to whether miR-186 served as an oncomir or a tumor suppressor miRNA in these specific cancers. We explored the possible mechanisms of the converse function of miR-186. We proposed that targets abundance and dose-dependent effects may contribute to the contradictory role of miR-186. To some extent, it was not so satisfactory a conclusion due to a lack of direct experimental data.

The following aspects deserve more attention in future studies: (A) Previous small size samples might be insufficient to reach a consistency in heterogeneous cancers, so a large cohort of patients are needed to specialize the function of miR-186 in each subtype of a specific cancer. (B) More targets of miR-186 related in tumorigenesis and metastasis are needed to be verified, which may account for its diverse functions. (C) That the targets abundance and the dose contributing to the final function of miR-186 in cancers needs to be verified intensively. (D) Other factors may disturb the function of miR-186 which can't be ignored, such as SNP either in miR-186 or the 3'UTR of the targets. (E) The upstream molecular regulators of miR-186, which are responsible for the altered expression of miR-186, such as Lnc RNAs, methylation, and the acetylation stage of the promoter of host gene ZRANB2, need to be explored. (F) More study is needed for other dual functional miRNAs [such as miR-181a (85) and miR-23b (87) in cancers] before we develop diagnostic and therapeutic strategies for cancer treatment based on miRNAs.

## Author Contributions

All authors approved the manuscript. YX initiated the topic and wrote the manuscript. LG and SN made the figures and tables. QT revised the manuscript.

### Conflict of Interest

The authors declare that the research was conducted in the absence of any commercial or financial relationships that could be construed as a potential conflict of interest.
